# Supplementary material for: Evolution of hospitalized patient characteristics through the first three COVID-19 waves in Paris area using machine learning analysis
Source: PLoS One. 2022 Feb 22;17(2):e0263266. doi: 10.1371/journal.pone.0263266 (PMC8863256; doi:10.1371/journal.pone.0263266)
Supplement: Attachment — Submitted filename: Response to Reviewers.docx [file pone.0263266.s001.docx]

November 18^th^ 2021

Dear Reviewers and Editors,

First of all, we greatly thank editors and reviewers for their careful reading, valuable advice and positive evaluations of our work.

Here are our responses for each review.

*Journal requirements*

- We based our manuscript on a PLOS One template for LaTex, including the *plos2015.bst* style sheet for BibTex. Still, we would gladly make any changes if needed.
- Code for model construction and validation was shared on a public GitHub repository so that anybody can check soundness and viability of the implementation.
- ORCID of the corresponding author was entered on the PLOS One editorial manager.
- Reference to Table 1 was added.

*Reviewer 1*

- Conflict of Interest section was modified so as to specify that indeed three of the authors are employees of Kaduceo, but the study did not receive any specific grant.
- As said above, code was shared on a public GitHub repository.
- Accuracy and AUC ROC Score were added for the Machine Learning model for each wave in a new table (Table 4), and also discussed in the Discussion section. Reported performances were rather low due to the low number of features used (age, gender and comorbidities). Nevertheless, the solid model construction and validation allowed to take into account the full discriminative power potential of the features to perform a robust and useful analysis of feature effects.
- Indeed our findings about the cardiovascular problem being a protective factor for the first wave is in contradiction with other studies. As written in the Discussion section, this could come from the fact the hospital in this study (CHI Créteil) does not have a large cardiology department, suggesting that some patients with heavy cardiovascular problems were redirected to other hospital centers because of the stressful context of the first wave.
- We provided a correct description of values (such as young or old age) along with the color indicator (blue or red).
- Original images (tiff format) respected the PLOS One Figure File Requirements. We guess it is the automatic PDF generator that blurred images, but we could provide image with a better resolution if still needed.
- Indeed this study was not large compared to some other studies, but covered a longer period of over one year. In consequence, we remove the word ‘large’ at the beginning of the Discussion section.
- We checked that the colormap used is colorblind friendly, for example using site such as ColorBrewer (https://colorbrewer2.org/#type=diverging&scheme=RdYlBu&n=10).
- References were added concerning ensemble tree models and their performances.
- Typos were corrected.

*Reviewer 2*

- Indeed, data was rather collected retrospectively from a prospectively maintained database. Abstract was modified in consequence.
- We clarified inclusion criteria in the Study population and design subsection in the Patients and Methods section. It consisted of every patient with a positive NAAT test or a chest tomography scan showing findings suggestive of COVID-19 disease.
- Despite the hospital of this study (CHI Créteil) being a large hospital center, it was not considered as a referral hospital of its area, notably because of the proximity of another larger center (CHU Henri-Mondor). Moreover, the CHI Créteil had a small Intensive Care Unit to be considered a referral hospital.
- French title of certain references were translated into English.
- We indeed changed ‘surveillance system’ into plural form.
- ‘Genetics factors’ was changed into ‘genetic factors’.
- In the Discussion section, we added some biological factors considered by the literature to have a significant impact on COVID-19 condition and aggravation, such as C-Reactive Protein (CRP) and Eosinophils. We also put appropriate references to support this claim.

Best Regards,

The authors
